# Therapeutic Ophthalmic Lenses: A Review

**DOI:** 10.3390/pharmaceutics13010036

**Published:** 2020-12-28

**Authors:** Nadia Toffoletto, Benilde Saramago, Ana Paula Serro

**Affiliations:** 1 Centro de Química Estrutural, Instituto Superior Técnico, University of Lisbon, Av. Rovisco Pais, 1049-001 Lisbon, Portugal; b.saramago@tecnico.ulisboa.pt (B.S.); anapaula.serro@tecnico.ulisboa.pt (A.P.S.); 2 Centro de Investigação Interdisciplinar Egas Moniz, Instituto Universitário Egas Moniz, Quinta da Granja, Monte de Caparica, 2829-511 Caparica, Portugal

**Keywords:** therapeutic contact lenses, therapeutic intraocular lenses, eye diseases, glaucoma, cataract, corneal diseases, posterior segment of the eye diseases

## Abstract

An increasing incidence of eye diseases has been registered in the last decades in developed countries due to the ageing of population, changes in lifestyle, environmental factors, and the presence of concomitant medical conditions. The increase of public awareness on ocular conditions leads to an early diagnosis and treatment, as well as an increased demand for more effective and minimally invasive solutions for the treatment of both the anterior and posterior segments of the eye. Despite being the most common route of ophthalmic drug administration, eye drops are associated with compliance issues, drug wastage by lacrimation, and low bioavailability due to the ocular barriers. In order to overcome these problems, the design of drug-eluting ophthalmic lenses constitutes a non-invasive and patient-friendly approach for the sustained drug delivery to the eye. Several examples of therapeutic contact lenses and intraocular lenses have been developed, by means of different strategies of drug loading, leading to promising results. This review aims to report the recent advances in the development of therapeutic ophthalmic lenses for the treatment and/or prophylaxis of eye pathologies (i.e., glaucoma, cataract, corneal diseases, or posterior segment diseases) and it gives an overview of the future perspectives and challenges in the field.

## 1. Introduction

A global increase in the number of people with an eye condition has been registered in the last decades, according to the WHO World Report on Vision 2019. While cataract remains the main cause of visual impairment in developing countries [1], the ageing of population increased the incidence of chronic posterior eye diseases (e.g., age-related macular degeneration (AMD)), glaucoma, and cataract [2]. Increased levels of air pollution, the prolonged use of steroids, and the increased incidence of allergic diseases are associated with disorders of the ocular surface, such as keratoconjunctivitis sicca or dry eye disease [3,4]. Other medical conditions, such as rheumatoid arthritis, multiple sclerosis, and diabetes, also present repercussions in the eye. Systemic microvascular damages that are associated to diabetes, in fact, affect both the anterior and posterior segment of the eye and they can lead to corneal issues, tear film instability, increased intraocular pressure, and a higher incidence of glaucoma, cataract, and uveitis, as well as pathologies of the back of the eye, such as diabetic retinopathy (DR) and diabetic macular edema (DME) [5]. Consequently, diabetic patients are 25 times more likely to become blind than the general population [6].

**Figure 1:**
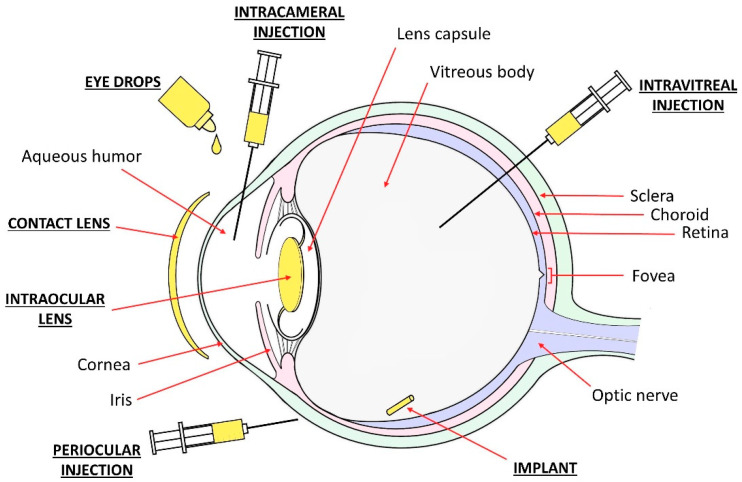
Ocular drug administration routes: eye drops, therapeutic contact lenses, therapeutic intraocular lenses, periocular injections (e.g., subconjunctival or sub-tenon injections), drug-eluting implant, intravitreal, and intracameral injections.

The public awareness on eye diseases is increasing, leading to an early diagnosis and the treatment of ocular pathologies as well as an increased demand for more effective and patient-friendly solutions. The local treatment of the eye is advantageous relative to systemic drug administration, since it avoids high concentrations of drug in the blood circulation and eventual undesirable side effects in other organs. Among the several possibilities for ocular drug administration (Figure 1), eye drops are the easiest route for topical delivery. However, this methodology is associated with compliance issues, especially in the case of elderly patients, who could have a limited ability to strictly adhere to the prescribed treatment [7]. After cataract surgery, for example, complex prophylaxis regimes are often adopted: it is not uncommon to prescribe a combination of three different drugs that are to be administered in multiple drops, several times a day, for a few weeks [8]. It was estimated that cataract surgery patients only apply half of the prescribed number of drops [9]. Compliance problems are also registered in glaucoma patients, as multiple formulations of topical drops for several years are commonly needed [10]. Other issues are the improper administration of topical medications, such as drops missing the eye, the delivery of an incorrect number of drops, and the contamination of the bottle tip [8]. Moreover, in order to avoid drug wash-out, it is suggested to wait 5 min. between two subsequent topical applications, but less than half of the patients complies with this recommendation [11]. Concerns also arise from the drug wastage by lacrimation and systemic absorption when topical administration is performed. The instillation of drops at fixed intervals causes a high variability of the intraocular drug concentration during the therapeutic treatment, which would be avoided with a continuous drug release. All of this, combined with the limited ability of the cornea to absorb drugs (at most, 5% bioavailability in optimal conditions [12,13,14], e.g., moderately lipophilic and moderately charged drugs, low molecular weight, the presence of permeation enhancers in the topical formulation [13,15,16]), contributes to the low efficiency of eye drop administration [17]. Ocular injections (e.g., intravitreal, intracameral, subconjunctival, and sub-tenon injections) are considered an alternative route to increase the efficacy of drug delivery, in particular to the posterior segment of the eye. However, injections lead to an initial high peak of ocular drug concentration followed by rapid decay [18,19]. While the use of biodegradable drug-eluting intraocular implants could overcome this issue and sustain drug release for several months, any form of ocular injection or ocular implant remains invasive for the patient and it is associated to a higher risk of adverse events when compared to eye drops [20].

In the last decade, increasing interest has grown toward to the possibility of designing drug-eluting ophthalmic lenses able to guarantee a sustained drug delivery to the eye in a non-invasive manner. Drug-eluting contact lenses (CLs) may avoid the frequent administration of eye drops and, therefore, increase patient’s comfort and compliance. By increasing the drug residence time on the cornea, the drug bioavailability is increased, while the necessary drug dose for achieving a therapeutic effect is reduced. Consequently, a lower systemic drug absorption is expected when compared to eye drops [21,22]. On the other hand, drug-eluting intraocular lenses (IOLs), implanted e.g., during cataract surgery, allow for overcoming the corneal permeability barrier by delivering drug directly into the aqueous humor, and sustaining drug release for weeks after surgery [23,24]. While CLs can be often replaced and are, therefore, suitable for both short-term and prolonged treatments, drug-eluting IOLs are a one-time treatment for the management of acute symptoms (associated, for example, with ocular inflammation after surgery [25,26]) or for the temporary replacement of the frequent and invasive intraocular drug injections to which chronic patients are often subjected [27].

Concerning CLs, patient compliance with good wear practices is often referred as a concern. The difficulty to place the CL in the eye, the eventual discomfort, the need of lens care and hygiene practices, and the need of fulfilling lens wear and/or replacement schedules are pointed to as the main reasons for the lack of compliance. However, a significant expansion of the number of wearers worldwide has been observed in the last two decades, nowadays reaching over 150 million people [28,29]. The evolution on the production technology of these devices, that turn them more comfortable, the implementation of a better patient’s education from the start of the lens-wearing and a close monitoring by practitioners were critical factors to enhance the adhesion to CLs. Moreover, the perception that the CLs are a prescribed medical device, and not just a commodity, and that they can be used for ‘treatment’, significantly increased the openness to their use. The previous use of CLs for refractive error correction, with all the knowledge of the required associated procedures, shall facilitate the acceptance of these devices as drug delivery vehicles among the patients.

The main goal of this review is to gather information on therapeutic ophthalmic lenses that have been developed in the last 10 years. First, the different drug loading methods adopted to tune the release profile of the lenses will be addressed. Subsequently, a vast number of examples will illustrate the potential of these innovative non-invasive drug delivery devices for the treatment and/or prophylaxis of glaucoma, cataract, corneal diseases, or posterior segment diseases. Finally, an overview of the future perspectives and challenges in the field will be given.

## 2. Methodology

The Scopus electronic database was consulted on 15 April 2020. The following search terms were used: lens AND drug release AND diabetic eye; lens AND drug release AND glaucoma; lens AND drug release AND diabetic cornea OR keratopathy OR dry eye OR keratitis OR edema OR keratoconus OR corneal dystrophy; lens AND drug release AND cataract prevention OR endophthalmitis OR inflammation OR infection OR PCO OR posterior opacification; and, lens AND drug release AND back of the eye OR posterior segment OR retina OR retinopathy OR vitreous OR macula.

Journal articles describing therapeutic ophthalmic lenses and published after 1 January 2010 were collected and analyzed. Only results that were published in English language were considered.

## 3. Drug Loading Methods

**Figure 2:**
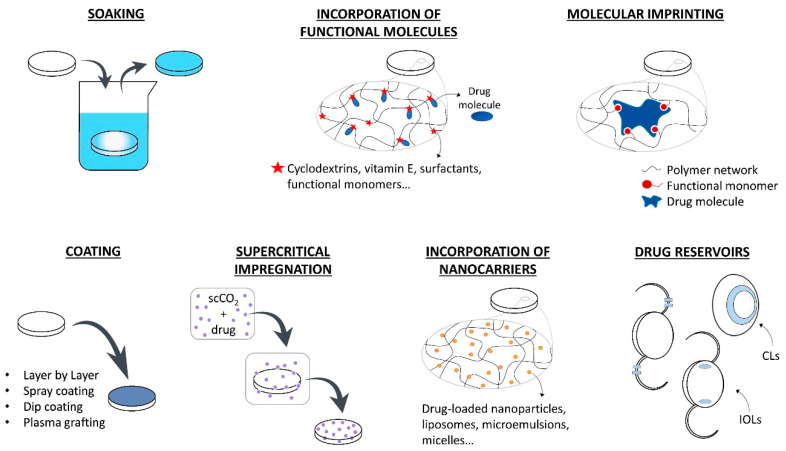
Strategies for the development of therapeutic ophthalmic lenses: soaking into a drug solution, incorporation of functional molecules with a high affinity to the drug, molecular imprinting, drug-eluting or drug-barrier coating, supercritical impregnation, incorporation of nanocarriers and incorporation of drug reservoirs.

In the following section, the various strategies that have been developed for loading drugs in therapeutic lenses are summarized. In Figure 2, a schematic description of these methods is presented.

### 3.1. Soaking

The simplest method for obtaining therapeutic lenses is soaking into a drug solution [24,30,31,32,33,34,35,36,37,38,39,40,41,42,43,44,45]. The amount of drug loaded and released depends on the material and the structure of the lens (e.g., porosity, swelling capacity), on the drug characteristics (e.g., molecular structure, molecular weight, charge), and on eventual interactions that may be established between the drugs and the lens material [22]. Besides, the soaking parameters also affects drug loading, i.e., the concentration of the solution [46], loading time [40], and environmental factors, such as temperature and pH [47]. The main issue of the soaking method still is the limited control over the drug release profile, which is usually characterized by a high initial release rate and a short delivery time after lens placement onto the eye [47,48]. Furthermore, economic and environmental concerns rise due to the waste of the drug present in the soaking solution [46]. Most drug-loaded lenses that are obtained by soaking are able to retain their therapeutic effect for a few hours or for some days [48], although a few examples of sustained release over weeks do exist [24]. This fast release kinetics could be suitable in the case of disposable CLs with a daily use, but it is not compatible with the development of IOLs with a long-term therapeutic purpose [49].

### 3.2. Incorporation of Functional Molecules

The incorporation of functional molecules into the lens polymer (e.g., cyclodextrins, vitamin E, surfactants, functional monomers) proved to be a potential strategy for enhancing drug loading during the soaking step and tuning the release kinetics. Cyclodextrins present a hydrophobic cavity that is suitable for accommodating hydrophobic drugs. They have been successfully co-polymerized with the lens backbone material to control drug delivery over time [25,50], or mixed to the drug solution prior to lens soaking in order to enhance the apparent aqueous solubility of hydrophobic drugs [51]. Vitamin E is considered to be a promising molecule for providing a hydrophobic diffusion barrier for the release of hydrophilic drugs [52,53,54,55,56]. When hydrophobic drugs are involved, drug molecules diffuse through the highly viscous vitamin E agglomerates, resulting in a slower release kinetic [57]. Because of the hydrophobic nature of vitamin E, it can be easily incorporated into the lenses by soaking in a vitamin E-ethanol solution. After ethanol evaporation, vitamin agglomerates remain trapped into the polymer network. However, a loss in oxygen permeability, protein adsorption, and changes in the mechanical properties can be associated with the use of this functional molecule in therapeutic lenses [22]. Aggregates that are composed of long-chain surfactants and oppositely charged ionic drugs can be added to the lens pre-polymer mixture to extend drug release over time by entrapment of the drug molecules [58]. The suitability of the lens polymer network to load and release drugs in a sustained fashion can also be improved by co-polymerization with functional monomers presenting a stronger affinity with the target drugs [59,60,61] or by the modification of the lens charge [62,63].

### 3.3. Coating

Several methods have been suggested to produce coatings on drug-eluting biomedical devices (e.g., layer-by-layer deposition, spray coating, dip coating, plasma-assisted grafting) with the purpose of implementing drug-eluting reservoirs [64,65,66,67,68,69,70,71,72,73]. Besides, these coatings may be used in order to increase the hydrophilicity of the device surface [74] and, also, as diffusion barriers to drug release [75,76,77]. The main concern with the design of coatings to be applied onto CLs or IOLs is the preservation of the optical properties of the original lenses. In the case of coatings with biodegradable polymers, the degradation products must be biocompatible with the surrounding tissues. Furthermore, the coating adhesion to the lens material should be sufficient for avoiding the presence of floating debris on the cornea or in the anterior chamber, which results from the coating detachment from CLs or IOLs, respectively.

### 3.4. Molecular Imprinting

Molecular imprinting can be obtained by two different methods, which involve either covalent or noncovalent bonds (e.g., ionic, hydrophobic, hydrogen bonds) between the template drug and functional monomers. The latter method is the most widely used in drug delivery, as it is associated to an easier and faster drug dissociation kinetics. Stable noncovalent drug-monomer complexes are established by solubilizing both the template drug and the functional monomers in the prepolymer mixture [78]. With the subsequent polymerization step, the functional monomers are covalently bonded to the polymer backbone, but not the drug, due to the presence of aromatic rings or other highly stable molecular structures that are less prone to react. The drug is removed during the washing cycles to which the newly polymerized biomaterials are subjected to eliminate potentially toxic unreacted monomers [79]. After drug removal, tailored memory sites remain imprinted in the polymer and, by mimicking the drug’s natural receptors, they create an oriented functional material with high affinity to the template drug [80]. The drug is then re-loaded in the polymer by soaking and interacting with the memory sites. This process can lead to a higher drug loading and a slower release profile [81], and it was successfully applied to therapeutic CLs and IOLs [82,83,84,85,86,87,88]. The use of miscible combinations of drugs and monomers is generally preferable over non-miscible ones in order to avoid the use of solvents, since their presence could prevent the orientation of monomers around the drug molecules and result in a less effective imprinting [89]. The drug affinity and the release kinetics are determined by the type of functional monomers and their concentration in the matrix: the ratio between functional monomers and drug molecules must be optimized for each combination of drug and monomers [22,89,90,91]. This ratio has to be sufficiently high to create enough interactions and retard drug release; however, an excessive monomer concentration could interfere with drug diffusion during the loading phase and could also impede an organized orientation of molecules, thus hindering any effect on the template drug. 

### 3.5. Supercritical Impregnation

Supercritical fluids are characterized by a high density, low viscosity, high diffusivity, and low interfacial tension [92]. These features are favorable for the diffusion of such fluids in the polymer matrices of CLs or IOLs [26,60,92,93,94,95,96,97]. Impregnation with a supercritical fluid consists in the dissolution of the drug in the solvent (generally supercritical CO_2_), followed by the interaction with the target material [22]. Supercritical CO_2_ is a better solvent for drugs than water, and also a better plasticizer for polymer networks: therefore, drug loading is enhanced when compared to traditional techniques like soaking [46]. This method is considered a ‘green’ alternative, as it forces the impregnation of the drug in the lens without using organic solvents. Moreover, CO_2_ is spontaneously released from the lens after impregnation, avoiding the purification steps that are usually associated to the use of solvents [92]. An optimized loading and release can be obtained by tuning the processing parameters, like pressure, temperature, presence of a co-solvent, duration of impregnation, and depressurization rate [22,46]. The choice of suitable parameters is fundamental: a too rapid depressurization, for example, can damage the lens material (foaming phenomenon [26]) and, therefore, compromise the optical functionality of the device. However, a too slow depressurization limits the efficacy of impregnation, as it reduces drug deposition into the polymer network [98]. The complex set-up is a limitation in the use of supercritical impregnation, as compared to other drug-loading techniques, and it can hinder its incorporation into the manufacturing process [89].

When applied to IOLs, supercritical impregnation allowed obtaining a sustained drug release over several weeks with various types of drugs (e.g., dexamethasone sodium and ciprofloxacin [26,92,95], methotrexate [97], cefuroxime sodium, and timolol maleate [98]). However, when the technology was applied to the thinner CLs, an initial burst release was commonly detected, followed by a sustained release for a few hours [93,94,99]. In order to overcome this issue, the combination of molecular imprinting and impregnation demonstrated to be an interesting alternative for the achievement of a gradual release, even in the first hours of CL wearing [100]. 

### 3.6. Incorporation of Nanocarriers

Colloidal nanocarriers entrapping active substances (e.g., polymeric biodegradable or non-degradable nanoparticles [74,101,102,103,104,105,106,107,108], liposomes, microemulsions, micelles) can be easily incorporated into the polymeric matrix of the lens [22]. Most of the nanoparticles for drug-delivery (homogeneous nanospheres or heterogeneous nanocapsules) are biodegradable polymers, which can release the drugs by degradation. Other mechanisms, such as light-induced release, can be implemented on non-degradable carriers [109]. Changes in pH and temperature, drug diffusion through the particle core/shell and matrix swelling also influence the drug delivery profile [110]. Liposomes have a structure that is similar to the biological membranes, in which lipophilic drugs can be loaded into the phospholipid bilayer and hydrophilic drugs into the aqueous core [111]. Polymeric micelles are spontaneously formed structures with promising applications in the delivery of hydrophobic drugs [112,113]. Microemulsions increase the drug-loading capacity during soaking and they present good thermodynamic stability, while preserving the ease of fabrication of the therapeutic lenses [114,115,116]. 

Despite the advantages of these innovative systems, significant problems need to be overcome during their design as drug-carriers for ophthalmic applications. In fact, changes in the mechanical properties and water content can be associated to their use in hydrogels [22]. Moreover, their natural tendency to aggregation must be hampered in order avoid a decrease in transparency [10]. 

### 3.7. Drug Reservoirs

Drug-eluting ocular implants have long been used as drug delivery platforms. However, their eventual migration after injection in the eye is one possible complication. The physical link of these implants to IOLs may overcome the problem and guarantee their correct positioning in the anterior chamber of the eye. Despite being more commonly applied on IOLs [23,27,117,118,119], examples of incorporated reservoirs were also implemented on CLs [120,121,122,123,124,125,126]. Drug reservoirs are usually constituted of biodegradable polymers: the material must be biocompatible, and its degradation products should not cause chronic inflammation or toxicity in the ocular tissues [47]. Reservoirs guarantee a prolonged (usually months) and controlled release over time. Moreover, the optical properties of the lens are not compromised, as they can be attached to the haptics of IOLs or incorporated into the peripheral part of CLs [12]. On the other hand, the design and fabrication of reservoirs can be complex and the physical link with the lens might be difficult to manage [46]. In the case of IOLs, the presence of these reservoirs can also raise problems while loading the lens in the injector system prior to surgery or in the ejection process [47].

## 4. Lenses for Ocular Diseases

### 4.1. Glaucoma

Glaucoma is the leading cause of irreversible blindness worldwide, and it is characterized by a progressive visual field loss due to the degeneration of retinal ganglion cells and optic nerve changes [5,127]. Although the relationship between diabetes and the development of glaucoma is still controversial and further investigation is needed, recent studies [128,129] have shown a significantly higher intraocular pressure (IOP) in diabetic patients when compared to non-diabetics, possibly due to an impaired autonomic function [129] and the progression of microvascular injury that is associated with chronic hyperglycemia [130]. An elevated IOP is considered to be a major risk factor for glaucoma, as it is associated to retinal ischemia and mechanical stress, as well as to an impaired ocular blood flow to retinal neurons [128].

The current treatment of glaucoma involves the lowering of IOP by pharmacological administration, laser, or surgical procedures [131]. The balance between the aqueous humor production and outflow from the anterior chamber of the eye regulates the IOP [128]. Therefore, current drug treatments are targeted at the reduction of aqueous humor production and/or the increase of the outflow facility. This can be achieved by use of beta-adrenergic antagonists (e.g., timolol, betaxolol, puerarin), sympathomimetic agents (e.g., epinephrine), parasympathomimetic miotic agents (e.g., pilocarpine), carbonic anhydrase inhibitors (e.g., dorzolamide), or prostaglandin analogues (e.g., latanoprost, bimatoprost) [132].

Topical drug application using eye drops is the first-line treatment [133]. However, an accurate drug administration is required in order to maintain a constant physiological IOP (generally 10–21 mmHg) [134]. Because of the chronic nature of the pathology and the reported poor patient adherence to the therapy [133], new drug delivery methods have recently attracted increased interest, including drug eluting CLs (Table 1).

Drug loading by soaking the material in a drug solution has been widely investigated for the treatment of glaucoma with therapeutic CLs [30]. In particular, different strategies were tested in order to increase the drug solubility in the loading solution and the drug amount loaded into the devices. Xu *et al.* [61], for example, co-polymerized *n*-vinylpyrrolidone (NVP), a hydrotropic agent, into pHEMA-based CLs in order to enhance puerarin solubilization into the hydrogel during the soaking phase. A six-fold increased residence time of puerarin in a rabbit eye model was obtained from NVP-modified CLs, as compared to traditional eye-drops. Xu *et al.* [115] and Wei *et al.* [116] suggested the use of microemulsions as soaking solutions to enhance the loading of bimatoprost and timolol, respectively: drug release was sustained up to 96 h *in vitro*, and a prolonged reduction of the IOP was observed in rabbits. García-Fernández *et al.* [51] evidenced that the incorporation of ethoxzolamide, a hydrophobic carbonic anhydrase inhibitor, in poly-cyclodextrins carriers enhanced the drug solubility in the aqueous soaking solution and its loading into HEMA-based CLs, while leading to a controlled release profile *in vitro*. Similarly, the addition of gold nanoparticles (GNP), either in the soaking solution or the polymer composition, resulted in a higher amount of timolol loaded into HEMA-based lenses. The presence of GNP in the lenses also augmented the drug deposition in the ciliary muscle of rabbits, where the majority of beta-receptors are located [107]. The time waste during drug loading is one of the disadvantages of the soaking method. However, Horne *et al.* [38] recently suggested a rapid soaking of commercial silicone hydrogel CLs (4 min.) into a non-aqueous solvent (*n*-propanol) to load latanoprost, a hydrophobic drug. *N*-propanol acted as a better solvent for the CLs than water, and the subsequent drug uptake correlated with the amount of lens swelling independently on the interactions between the lens material and the drug. Drug deposition in the lens and propanol removal was then obtained by washing in water, which is a non-solvent for the hydrophobic latanoprost. The release in artificial tear solution lasted over days.

The group of Chauhan used a pre-soaking in a vitamin E-ethanol solution, prior to soaking in the timolol-PBS solution, in order to form aggregates that have a barrier effect on drug diffusion from commercial silicone lenses [55,56]. In glaucomatous dogs, CLs containing vitamin E released timolol with the same efficacy of eye drops, but with reduced drug loss. The same group developed dual-drug delivering lenses [52] while using vitamin E presoaking: the simultaneous loading of timolol and dorzolamide increased the release duration of both drugs up to two days. A superior IOP reduction was observed with CLs in dogs, as compared to eye drops administration. Moreover, the effect on IOP lasted for one week after removal of the device, which is promising for future clinical applications. Vitamin E presoaking was also adopted in order to increase the delivery duration of bimatoprost up to 10 days *in vitro* [53]. Lee *et al.* [54] experimented the simultaneous loading of vitamin E or vitamin A and anti-glaucoma drugs (i.e., timolol or brimonidine) on hydrophilic HEMA-based CLs: the treatment enhanced drug loading, but no effects on the release kinetic were detected. Lee *et al.* did not provide an explanation for this behavior, but it may be related to the fact that vitamin E precipitates at the interfaces of the biphasic silicone material and the transport of the drug is a combination between diffusion in the aqueous phase and surface diffusion over the vitamin E aggregates [139]. In contrast, the monophasic HEMA material does not provide preferential sites for the adsorption of vitamin E agglomerates, which will not affect drug release.

In other cases, drugs were incorporated inside the lens material during the polymerization step while using micro or nanocarriers. A 48-h release *in vitro* was obtained by including timolol-loaded ethyl cellulose microparticles into the polymer mixture [135]. The addition of timolol-loaded PGT (propoxylated glyceryl triacylate) or EGDMA (ethylene glycol dimethacrylate) nanoparticles resulted in a sustained release *in vitro* for over 20 days, even after five months of packaging in refrigerator [101,102]. The incorporation of mPEG-PLA (methoxy poly(ethylene glycol)-poly(D,L-lactide)) micelles allowed for the simultaneous release of timolol and latanoprost for 120 h and 96 h, respectively, and a reduction of IOP for over 168 h in rabbits [113]. 

Molecular imprinted CLs were also successfully studied: uptake and release kinetics of timolol [82], bimatoprost [83], ethoxzolamide, and acetazolamide [84,85] were positively influenced by the presence of imprinted domains in the lens material, without significant affection of the CL properties. Timolol was released up to 90 h *in vitro*, due to the interactive sites between the drug and HEMA, carboxy-methyl chitosan, and acrylamide, which compose the CL material. Bimatoprost was released up to 36–60 h *in vitro*, and a low initial burst release was observed in rabbit tear fluid when compared to eye drops administration and non-imprinted drug-loaded CLs. Interestingly, Deng *et al.* [86] developed a structural-colored timolol-imprinted CL, which was able to shift from green to blue with timolol release, by using a monodispersed silica nanoparticles mold. Imprinting with methacrylic acid increased the loading amount and the residence time of the drug (up to 12 h), while the binding and unbinding of timolol molecules caused changes in the volume and refractive index of the material, which is translated into an optical signal for the monitoring of drug release.

Costa *et al.* [93,94] applied the supercritical solvent impregnation technology to the drug loading of commercial CLs: the amount of timolol maleate (TM) and acetazolamide loaded into the lenses was tuned by adopting different solvent mixtures and parameters, without alterations of the CL features. However, an initial burst characterized the release profile and it was not sustained over time.

More recently, the group of Ahmad [67] fabricated drug-loaded coatings on CLs while using various techniques: timolol-loaded PNIPAM (poly-*n*-isopropylacrylamide) and/or PVP (polyvinylpyrrolidone) coatings were obtained by electrohydrodynamic atomization, an electrically driven spraying process, and released TM for hours *in vitro*. Similar results were obtained by electrospinning PVP-PNIPAM-TM solutions incorporating corneal permeation enhancers [68]. In another study [69], chitosan and borneol were incorporated in PVP-PNIPAM-TM atomized coatings as a release modulator and a permeation enhancer, respectively. Chitosan also acted as a permeation enhancer, and increased TM release by up to 23%. However, in all cases, the presence of a mask during coating deposition was necessary to preserve the optical functionality of the central part of the lenses.

Examples of polymeric ring-shaped drug reservoirs have been designed to be separately fabricated and subsequently incorporated in CLs. The implantation of ethyl cellulose nanoparticle-laden rings [121] in HEMA/MAA CLs sustained TM release for 168 h *in vitro* and caused a significant IOP reduction in rabbits for 192 h. The dual-delivery of timolol and hyaluronic acid, a comfort agent, was obtained from HEMA/DMA-based rings [122] with comparable results: timolol was detected in rabbit tear fluid for 72 h, with an effect on IOP for 144 h. The same group also incorporated multiple reservoirs into the peripheral region of the CLs for the simultaneous delivery of timolol, bimatoprost, and hyaluronic acid [123]. Ciolino *et al.* [124,125] obtained a long-lasting release of latanoprost (one month), both *in vitro* and in a rabbit model, from PLGA spin-coated films that were implanted in the peripheral region of methafilcon CLs. However, as rabbits were not responding to the latanoprost therapy, the device was also tested on glaucomatous monkeys: the results evidenced that the IOP lowering efficacy was as least equal to the daily administration of eye drops. Song *et al.* [126] engineered multifunctional CLs, which were able to measure IOP, in order detect glaucoma biomarkers and deliver timolol for one month *in vitro*: an aluminum oxide nanoporous thin film with a central hole was incorporated in PDMS lenses, and served both as a power-free sensor and a drug reservoir with a prolonged release over time.

The management of storage time and conditions is another issue related to drug-loaded devices. In order to prevent drug loss during shipping and storage, triggered release systems have been proposed in the last years, and some strategies have been applied to CLs. Inner layer-embedded CLs with a pH-triggered release of betaxolol hydrochloride were obtained by Zhu *et al.* by incorporating the drug in a cellulose acetate/Eudragit S100 blend film. The drug was detected for 240 h in the tear fluid of a rabbit model [138]. Lysozyme-triggered degradation of acetylated chitosan was suggested for the release of TM in the presence of the lacrimal fluid enzyme [103]: briefly, nanogels were synthetized by crosslinking polyethyleneimine-coated nanodiamonds with chitosan in the presence of TM, and then incorporated into CLs. The degree of chitosan acetylation controlled the polymer degradation and, therefore, the release profile. A sustained and gradual release was detected for at least 48 h *in vitro*. A different strategy was used to obtain daylight-mediated timolol release. The drug molecule was linked to the CL by a photocleavable caged cross-linker (dimethoxy-substituted 2-nitrobenzene caged group) [136]. Bond breakage was triggered by 400–430 nm light, and therapeutic amounts of timolol were released for 10 h. The estimated relative low fabrication cost would also allow for a daily lens disposal and it would avoid risks of contamination during reloading procedures. A drug release that was triggered by body temperature was obtained while using PNIPAM as a carrier for TM: bicontinuous microemulsion CLs, constituted by continuous aqueous and oily phases, provided a nanoporous structure that was suitable to load a TM-PNIPAM nanogel by centrifuging and soaking [104]. The release time (72 h–30 days) was controlled by selecting the appropriate initial volume of drug loaded, the loading parameters, and the loading medium. *in vivo* experiments on rabbit models detected TM in the aqueous humor up to seven days, with a significant decrease of IOP within 2 h from lens application [137].

### 4.2. Cataract

A cataract is defined as the opacification of the natural crystalline lens with a consequent decrease in the quality of vision [140]. It is the leading cause of vision loss in elderly patients, causing visual impairment in about 30% of the population over 65 years old, but its incidence is also increasing in the younger population due to the exposition of UV radiation, smoke, use of steroids, increased incidence of diabetes and malnutrition [46,141]. Approximately 20% of cataract surgeries in the western population are performed on diabetic patients [142].

Diabetic crystalline lenses are characterized by an increased level of free radicals and impaired antioxidant capacity, which leads to an increased susceptibility to oxidative stress. Cataract development occurs at an earlier age in presence of diabetes, but an appropriate metabolic control can contribute to the lens recovery in young patients [143]. The administration of aldose-reductase inhibitors (ARIs) and antioxidants was also demonstrated to be beneficial in the delay of diabetic cataract in the early stage of the pathology and it could be useful for its prevention in patients at risk. In order to provide drug-eluting CLs for the local treatment of the diabetic eye, the ARI epalrestat was loaded into silicone hydrogels [88]. A biomimetic strategy was applied for the selection of the hydrogel composition. After loading by soaking, a strong affinity between the drug and aminopropyl methacrylamide (APMA)-functionalized hydrogels was observed and epalrestat was released for up to one week *in vitro*. The device successfully prevented opacification in extracted porcine crystalline lenses under hyperglycemic conditions [88]. Examples of antioxidants-eluting CLs have also been reported. Yigit and Ercal [37] soaked commercial CLs in *n*-acetylcysteine or *n*-acetylcysteine amide solutions and obtained a suitable release *in vitro* for daily use of the lenses. Varela-Garcia *et al.* [144] increased the uptake and the released amount of transferulic acid from hydrogels by functionalization with cytosine, a nitrogenous base with high affinity for a variety of drugs. Functionalization was performed by soaking the hydrogels in a cytosine/water/dioxane solution after polymerization.

Cataract surgery currently remains the standard treatment of severe cataractous eyes. The procedure consists in the removal of the damaged lens and a subsequent implant of an IOL. Despite the advances in the technique and the evolution in the different types of IOLs made this procedure one of the most cost-effective in the current healthcare [46], some post-operative complications can still occur and cause patient discomfort and visual impairment, as well as a prolonged recovery time [145]. An inflammatory response due to the physical trauma that is associated to surgery is considered to be physiological and it is generally treated with topical non-steroidal anti-inflammatory drugs (NSAIDs) or corticosteroids. If untreated, persistent inflammation can lead to pseudophakic cystoid macular edema (PCME), uveitis, iritis, glaucoma, and an increase of IOP [145,146]. Diabetic patients are at increased risk of developing these diseases. A recent clinical trial by McCafferty *et al.* [146] on 662 patients evidenced that PCME was the most common complication after cataract surgery. Because of the higher sensitivity of the vascular bed in diabetic patients, approximately 4–12% of patients affected by diabetes mellitus [142,147] and up to 56% of patients with diabetic retinopathy [148] are expected to develop PCME after IOL implant. Ocular infection, often linked to endophthalmitis, is another possible adverse condition, and it is mostly caused by bacterial migration from the lid and conjunctiva to the intraocular space. In this case, prevention and treatment involve the use of topical corticosteroids and antibiotics [145]. The migration and proliferation of epithelial cells to the posterior surface of the IOL can also lead to visual impairment after surgery; the phenomenon is known as posterior capsule opacification (PCO), and it is commonly treated by a laser procedure [149]. Postoperative endophthalmitis and early PCO are both reported to develop at a higher incidence rate in diabetic patients [150,151].

The current prophylaxis after cataract surgery consists in the administration of antibiotics and anti-inflammatory drugs (NSAIDs and/or corticosteroids) in the form of eye drops for two to four weeks after IOL implantation. The Food and Drug Administration approved a new intracameral drug-release device (DEXYCU, Icon Bioscience Inc., Newark, CA) in 2018 for the treatment of postoperative inflammation associated to cataract surgery [152]. The device is constituted by an injectable dexamethasone suspension, which is able to guarantee a 21-days long therapeutic level of drug release. The suspension is injected at the end of cataract surgery, forming a surface tension-based sphere positioned aside the IOL and behind the iris. The use of CLs as drug depots for antibiotics and anti-inflammatories has been proposed as a less-invasive alternative (Table 2) [21,111]. However, in the case of cataract surgery, postoperative prophylaxis could be more efficiently achieved by use of drug-loaded IOLs (Table 2), in order to overcome the issues that are related to patient compliance and poor drug permeability through the cornea, to which both eye drops administration and drug-eluting CLs are subjected [47].

By soaking in antibiotic solutions (i.e., moxifloxacin or gatifloxacin) for 15 min, Lipnitzki *et al.* [39] obtained a drug-eluting HEMA-MMA IOL that, when implanted into rabbit eyes, exhibited a slower decrease of the drug concentration in the aqueous humor as compared to a single intracameral drug injection performed after surgery. However, the drug could only be detected up to a few hours after implant placement. More recently, Topete *et al.* [40] reached a minimum of eight-day release period of moxifloxacin, diclofenac, or ketorolac from commercially available hydrophilic IOL material through the optimization of the temperature and time of soaking. In particular, loading at 60 °C for two weeks allowed for maximizing drug loading and sustaining moxifloxacin or diclofenac release over time. Subsequent *in vivo* studies on rabbits [41] proved the suitability of moxifloxacin-loaded IOLs as delivery devices at therapeutic drug concentrations for at least one week. In order to perform an effective prophylaxis for endophthalmitis after cataract surgery, the simultaneous release of moxifloxacin and ketorolac was then successfully experimented, obtaining the release of the antibiotic and anti-inflammatory drugs for at least 15 days. The presence of both drugs enhanced the release profile [24]. Pimenta *et al.* also achieved the dual release of NSAIDs and antibiotics (i.e., diclofenac and moxifloxacin) [42]. In this case, loading was sequentially performed in the two drug solutions. The obtained IOLs are expected to be effective *in vivo* for three weeks.

Li *et al.* [25] incorporated β-cyclodextrins into HEMA-MMA hydrogels in order to increase the amount of dexamethasone loaded during the soaking step in the drug solution. A sustained drug release was obtained through the hydrogel modification, but it only lasted a few days.

Supercritical fluid impregnation has also been applied to IOL materials. In 2012, González-Chomón *et al.* [60] developed an acrylic hydrogel that was based on the addition of 2-butoxyethyl methacrylate (BEM) to HEMA. This hydrogel was foldable even in dry state and therefore implantable through minor corneal incision. An increase in the BEM fraction led to a lower swelling due to the hydrophobic character of this compound and reduced norfloxacin loading by traditional soaking methods. This problem could be solved by supercritical CO_2_ impregnation that notably increased drug loading in the hydrogels. In the same study, the incorporation of small quantities of acrylamide or methacrylic acid as functional monomers was also tested with positive outcomes on the total amount of drug loaded. Bouledjouidja *et al.* loaded commercial foldable HEMA-based [26] or rigid MMA-based [92] IOLs with ciprofloxacin or dexamethasone. *in vitro* drug release was kept for several days by the optimization of the impregnation parameters (i.e., pressure, duration, and presence of a co-solvent). No effect on the dioptric power of the impregnated lenses was detected [95]. Ongkasin *et al.* recently adopted the same technique for the loading of gatifloxacin [96] into commercially available hydrophobic IOLs. The effect of the parameter optimization on the impregnation yield was evaluated, but no data on the drug release profile were reported.

In other studies, the application of either drug-eluting or drug-barrier coatings onto the IOL surface was attempted. Layer by layer (LbL) deposition was adopted as a drug-depot coating carrying ampicillin [65], and a sustained release was obtained over days. LbL coatings were tested as barriers to the release of diclofenac, moxifloxacin, ketorolac, or chlorhexidine [70], but only diclofenac evidenced a slower release after the modification. Plasma-assisted grafting with HEMA [71] or 2-acrylamido-2-methylpropane sulfonic acid [66] in the presence of moxifloxacin resulted in a 15-day drug release above the therapeutic concentration, which is comparable to the common duration of the prophylactic treatment that is required after surgery.

The incorporation of drug reservoirs in the implanted IOL was first suggested between 2006 and 2008 [27,117]. More recently, antibiotics, like norfloxacin [23] and levofloxacin [118], were loaded into polymeric reservoirs that were attached to the IOL’s haptics or to the lens edges, respectively, and released a clinically relevant drug dosage for at least 15 days. Two reservoirs containing different drugs could be paired in order to obtain a synergic effect: Eperon *et al.* [119] combined the release of triamcinolone acetonide and cyclosporine A to increase the therapeutic effect of the IOL. The implanted system reduced inflammation for more than three months in rabbits.

The incidence rate of PCO can be lowered through an appropriate design of the implanted IOLs. Several studies focused on the influence of the lens material [155], edge squareness, and surface modifications [156,157] in order to inhibit cell adhesion and proliferation on the device. Square edges, in particular, inhibit the migration of epithelial cells to the optic part of the IOL, thus preventing the pathology [158]. Drug-eluting IOLs have been recently proposed to pharmacologically hinder PCO. Celecoxib [32], erufosine [33], erlotinib [34], gefitinib [35], and methotrexate [36] were loaded with this purpose into IOLs by soaking in drug solutions. Despite the short-term release that is achieved with most of the investigated systems, a long-term effect on the proliferation of epithelial cells was registered with ex vivo canine models or human capsular bag models, suggesting the suitability of the method.

Drug-releasing PLGA coatings were designed for the release of rapamycin [72], methotrexate (MTX) [64] or Y27632, an inhibitor of Rho-associated kinase [73]: in the case of rapamycin, the coating was applied to the edge of the IOL optics, while MTX and Y27632-enriched coatings were applied to the optic part without affecting transparency. Rapamycin-loaded IOLs resulted in being more effective in treating PCO in rabbits as compared to the administration of capsular irrigation during surgery or eye drops, and the drug was detected in the aqueous humor for weeks. MTX-loaded IOLs showed promising results in a human capsular bag model: the drug was released for 14 days, but a high initial burst release was observed. Y27632 was released *in vitro* for only one day. Despite this, a significantly lower PCO incidence was registered in rabbits after implantation, in line with previous studies regarding the long-term effect of short-term treatment for PCO.

Zhang *et al.* [77] obtained the laser-triggered release of indocyanine green, a photosensitizer, from commercially available IOLs by spray-coating the active agent on the lens surface and, subsequently, controlling the release with a sealing layer of PLGA. However, low transmittance, related to both the presence of indocyanine green and to PLGA degradation, remained an issue over several weeks after immersion in PBS, affecting the suitability of the system for the intended ocular application.

Chitosan nanoparticles that were loaded with doxorubicin [74,105] or 5-fluorouracil [106], applied to the IOL surface by LbL deposition or ion-beam functionalization, also sustained drug release over days and reduced PCO incidence in rabbits.

Different matrix metalloproteinases inhibitors (MMPI) were embedded in a PDMS lens mixture prior to curing or by soaking the PDMS discs in a drug-ethanol solution for four days. For some of the obtained systems, drug release was sustained for up to five months and a significant reduction in the human epithelial cell migration rate was observed after *in vitro* exposure to the drug-eluting lenses [154].

Supercritical fluid technology was also suggested to implement methotrexate releasing acrylic IOLs. The drug was eluted *in vitro* for more than 80 days. In this case, the device did not cause any significant difference in the time that is required to reach cell confluence in a human capsular bag model, but fibrosis was reduced by inhibiting cell transformation from epithelial to mesenchymal phenotype, which has a major role in PCO formation [97].

### 4.3. Corneal Diseases

Keratoconjunctivitis sicca, or dry eye syndrome, is a common condition of the anterior segment of the eye, which can be associated to discomfort, burning sensation, external body sensation, ocular pain, light sensitivity, and intermittent blurred vision [159]. The presence of a normal tear film is fundamental for corneal health and immune protection [160] and the prevention of corneal injuries. Ocular lubrication can be compromised by reduced tear production and/or instability of the tear film, leading to a rapid evaporation from the eye surface. Various causes have been identified, including environmental factors (low ambient humidity, excessive wind or dust, temperature extremes, air conditioning), ageing, allergies, a prolonged computer use, metabolic conditions and nutritional deficiencies, contact lens use, and the prolonged administration of systemic or topical drugs (e.g., antiglaucoma medications or preservative-containing eye drops) [161]. Patients that are affected by neuropathic disorders, Sjögren’s syndrome, lupus, blepharitis, or congenital abnormalities of the lid are more prone to developing dry eye symptoms [162].

Other common corneal diseases are keratitis, a corneal inflammation that is usually treated by topical or systemic administration of antibiotics, biocides, antifungals, or antivirals, depending on the type of infection [163], and exposure keratopathy, often caused by an inadequate eyelid closure and treated by the use of lubricating drops or by surgical procedure [164]. Bullous keratopathy and Fuch’s dystrophy, on the contrary, are caused by an impairment of the corneal endothelium which loses the ability to drain fluid out of the cornea. Hypertonic topical solutions are usually prescribed to reduce the resulting corneal edema [165], and the concomitant wearing of bandage contact lenses is suggested to increase the residence time of eyedrops on the corneal surface [166,167]. Inherited corneal dystrophies, such as keratoconus, lattice dystrophy, and map-dot-fingerprint dystrophy, can lead to a major visual impairment at their advanced stage [168].

Although often overlooked, complications in the anterior segment of the eye are also common in diabetic patients [169]. It is estimated that diabetic keratopathy affects approximately 47–64% of diabetic patients and, if not treated, it can lead to major visual impairment [170]. Keratopathy is associated with an increased risk of corneal epithelial defects, recurrent erosions, delayed epithelial wound healing, tear film alteration, edema, and neurotropic corneal ulcers [160,169,170]. In severe cases, hyperglycemia and microvascular damage cause corneal neurotrophic lesions and a progressive decrease in corneal nerve fiber density, which lead to a loss of sensitivity, an impairment of the epithelial healing process, and a lack of feedback control over tear secretion [169,171]. An abnormal regulation of the healing mechanism can cause corneal opacity and blindness [172]. Despite the suggested causality between peripheral neuropathy and diabetic keratopathy, direct alterations in the corneal epithelium were also observed in less severe conditions, without signs of neuropathy [160]. In the early stage of the pathology, diabetic patients frequently experience dry eye symptoms [171]. Hyperglycemia, in fact, can cause a microvascular damage to the lacrimal gland, also contributing to the low tear secretion [169]. Alterations in the tear film were also associated to the presence of inflammation, oxidative stress, and the accumulation of advanced glycation end products (AGEs) in the lacrimal gland [160]. Tear film changes, such as a reduced lipid layer quality and film stability, were also registered in diabetics [173].

The current topical treatment of the dry cornea pursues the objective of maintaining a lubricated ocular surface and an intact epithelium through tears replacement [174]. Artificial tears are helpful in maintaining a healthy ocular surface and clear vision [169]. However, they have a low residence time (5 min.) and need to be administered more than nine times a day, in most serious cases of dry eye [175,176]. CLs with incorporated moisturizing macromolecules (Table 3) constitute a strategy for increasing the patient comfort and prevent dry-eye symptoms that are associated to the continuous CL wear. Moreover, their use can avoid periods of blurred vision being encountered after the administration of lubricating eye drops. Several examples of daily disposable lenses with immobilized or free moisturizing agents are already available on the market, such as Focus Dailies with AquaRelease (Alcon), Dailies AquaComfort Plus (Alcon & Ciba Vision), 1-Day Acuvue Moist (Johnson & Johnson), and Fusion 1 day (Safilens). The release of comfort agents, as compared to immobilization onto the CLs, presents the advantage of distributing the effect on the ocular surface even away from the lens and increasing the agent effectiveness [81]. Research has been focused on the optimization of a sustained release over time of such macromolecules. Several examples have been reported, such as the incorporation of polyvinyl alcohol (PVA), polyvinylpyrrolidone (PVP), or hyaluronic acid (HA), widely used in artificial tears, into soft CLs for the relief of dry eye symptoms [175]. More recently, Maulvi *et al.* investigated different loading methods for HA on CLs: soaking, direct entrapment during polymerization, and a HEMA-HA ring reservoir. Co-polymerization with HA allowed a sustained delivery for 15 days in rabbit eyes [176], while a nine-day release was obtained from the ring-CLs system [177]. For the same purpose, and through molecular imprinting on silicone hydrogel CLs, White *et al.* [81] managed to design the release of hydroxypropyl methylcellulose (HPMC), a re-wetting agent, up to 60 days in dynamic release conditions. The same strategy was applied by the group in order to reach the simultaneous delivery of both re-wetting and anti-inflammatory agents (i.e., trehalose, HPMC, prednisolone, and ibuprofen) [178], which, even if not specified by the authors, could be interesting to address the multiple complications of the diabetic cornea. Acrylic acid and 4-vinylphenol were selected as functional monomers, and the molecules were released for several hours *in vitro*. It is worth considering that, in several pathological eye conditions, as in the case of dry eye, the tear pH is also affected. Based on this, Kim *et al.* [179] suggested a pH-triggered release of HPMC from smart HEMA-PVP or HEMA-PNIPAM CLs. HPMC was loaded by soaking, and a release over hours was observed in cyclic pH conditions. Another approach is to directly address the improvement of the tear lipid layer quality, whose instability (that is commonly observed in diabetic dry eye) causes a rapid evaporation and decreased lubrication. With this aim, Pitt *et al.* [180,181] engineered the daily release of phospholipids from commercial silicon hydrogel CLs through *in vitro* studies. The loading of phospholipids was performed by rapid soaking (30–120 s) in a *n*-propanol solution. The optical transmission and wettability of the lens were not affected by the process. The optimization of the loading parameters (i.e., loading solution concentration, loading time) subsequently allowed achieving a sustained release for 30 days from reusable CLs *in vitro* [182]. The use of osmoprotectants could also contribute to the reduction of hypertonic stress damage, as dry eye syndrome in diabetic patients is accompanied by hyperosmolarity of the tear film [183]. A step in this direction is represented by the study of Hsu *et al.* [184], who loaded betaine, an osmoprotectant, or dexpanthenol, a moisturizing agent, into silicon-hydrogel CLs. A pre-soaking in a vitamin E solution was performed prior to soaking in betaine or dexpanthenol solutions, in order to increase the *in vitro* release duration time from approximately 15 min. to 12 h.

Although the administration of lubricating agents can contribute to symptom relief and the prevention of corneal injuries, a pharmacological treatment is often prescribed in pathological conditions (Table 3). The use of topical antibiotics and anti-inflammatory drugs is suggested to alleviate surface inflammation and promote the re-epithelialization of the cornea [169,174]. The most widely used anti-inflammatory topical drugs for dry-eye syndrome are corticosteroids, NSAIDs, and cyclosporine A [185]. In recent years, several examples of the mentioned drugs have been incorporated into CLs using many different techniques, and the obtained results have been extensively discussed in previous review papers [21,111,186]. Therapeutic CLs for the treatment of keratitis have also been widely reported in literature reviews [187,188], with promising results for the delivery of biocides [189,190], antifungals [189,191,192,193,194], and antiviral drugs [195,196].

In 2014, Jacob *et al.* proposed a CL-assisted surgical treatment of keratoconus [197]. The loss of structural stability associated to the pathology results in a progressive corneal thinning, deformation, and impaired vision. The structural support of hard contact lenses is the first-line treatment, but their use does not alter the progression of the condition. The photo-induced collagen polymerization emerged in the last decade as an effective method for stopping the progression of keratoconus and increase the corneal stiffness [198]. In order to perform the procedure, riboflavin is used as a photosensitizer for collagen. By soaking a commercial UV barrier-free CL into a riboflavin solution, it was possible to obtain a riboflavin reservoir for corneal crosslinking. The riboflavin-CL system also artificially increased the thickness of the lens-corneal layer, which results in a UV protective shield for the corneal endothelium. Pilot studies were conducted with promising results [199].

Overall, the controlled use of topical corticosteroids or NSAIDs can be beneficial in inflammatory corneal diseases. However, it must be stressed that the prolonged use of steroidal drugs is associated to an increased IOP and a higher incidence of cataract, for which certain categories, such as diabetic patients, are already considered to be at risk. On the other hand, NSAIDs can lead to a decrease in corneal sensitivity and the dissolution of corneal epithelium [185]. Even in absence of adverse events, their application remains limited to the treatment of dry eye-related symptoms. Promising new strategies have been suggested for a wider approach to the anterior segment pathologies, addressing, for example, neurotrophic keratitis, persistent epithelial defects, diabetic keratopathy, and diabetic neuropathy, such as the use of topical insulin, naltrexone, ARIs, substance P, and different growth factors [160,169,170,174,200].

Naltrexone (NTX) is an opioid antagonist, whose systemic or topical administration proved to block the negative effect of opioid growth factors, which are excessively present in diabetic patients, on cell proliferation and tissue growth. The use of naltrexone eye drops resulted in an accelerated corneal wound healing and the restoration of corneal sensitivity in many animal models, and it is considered to be a promising treatment of diabetic keratopathy [160,174]. Alvarez-Rivera *et al.* recently developed a NTX-releasing CL [87] while using molecular imprinted HEMA hydrogels. A bioinspired approach was applied for the selection of functional monomers and the incorporation of acrylic acid was found to increase the amount of drug that was loaded into the hydrogel. NTX was released from imprinted CLs in a sustained fashion for at least two days in both sink and dynamic conditions *in vitro*, and the attained drug concentration in a bovine cornea model was comparable to that obtained with administration of a drug solution (t = 6 h after administration).

Several growth factors (i.e., epidermal growth factor, basic fibroblast growth factor, transforming growth factor-beta, platelet derived growth factor, insulin-like growth factor, and vascular endothelial growth factor) have a role in the control of corneal epithelial cells proliferation, migration, and apoptosis, and they are fundamental in the corneal healing process [203]. The topical administration of growth factors proved to be beneficial in the treatment of persistent epithelial defects, neurotrophic keratitis, and for the recovery of corneal epithelium after surgery [200]. Altered levels of growth factors have been also registered in diabetic tissues, and the restoration of their physiological expression has been tested with promising results in clinical trials [160]. The release of growth factors from CLs was also investigated, aiming to increase the bioavailability and residence time of such therapeutic agents. In 2010, Schultz and Morck [43] obtained vasurfilcon A CLs (that are composed of methyl methacrylate, vinyl pyrrolidone, and other methacrylates) that are able to release epidermal growth factor (EGF): after loading in a EGF solution for 7 h, EGF was released for 4 h *in vitro*. Despite the short duration of release, a therapeutic effect of the obtained device was observed on rabbit eyes with induced corneal abrasion as compared to untreated eyes. The same EGF loading procedure resulted unsuitable for silicone-based CLs. Later, the same group performed preliminary clinical tests by applying the EGF-loaded CLs onto nine patients with corneal epithelial defects not responding to conventional therapies [202]. Complete recovery was achieved in seven patients after wearing the device for 4–13 days. A previously developed significant ocular inflammation was reported in the two unsolved cases. No adverse events were registered, but wider randomized and controlled clinical trials are still requested in order to assess the efficacy of the device and, in particular, its suitability for the treatment of metabolic-induced corneal defects. Pursuing the same objective, Sandri *et al.* [203] loaded platelet derived growth factors (PDGF) onto various commercial CLs. In order to perform the loading, two solutions of platelet lysate and chondroitin sulfate were poured into the concavity of the CLs and then kept for 24 h at 25°C. Due to its electrostatic interaction with positively-charged growth factors, the presence of chondroitin sulfate stabilized and reduced degradation in PDGF. After loading, PDGF were gradually released over 6 h from PureVision^®^ silicone lenses *in vitro*, while promising results were obtained by preliminary *in vitro* wound healing assay.

The administration of ARIs proved to successfully inhibit the polyol pathway, being responsible for several diabetic eye complications, including dry eye syndrome. Both oral and topical treatment with ARIs improved tear production and corneal sensitivity, reduced nerve damage, and promoted corneal epithelial regeneration in diabetic patients [169,185,204]. Therefore, the previously mentioned incorporation of epalrestat into silicone CLs [88] could perform a multiple action for a wider control of diabetic-related ocular pathologies, including keratopathy, neuropathy, and cataract prevention. Similarly, the administration of topical antioxidants can not only be beneficial for the symptoms of retinal diseases, glaucoma, and diabetic cataract, but also for keratopathy and dry-eye syndrome [144,205].

Tissues in the anterior segment of the eye are particularly at risk for the development of atmospheric and light-induced oxidative stress. In physiological conditions, several defense mechanisms are active in preventing damage, such as the presence of antioxidant agents in the tear fluid and in the aqueous humor. Ascorbic acid, lactoferrin (LF), uric acid, and cysteine [205], for example, are present in high concentration in the tear fluid, but, in the case of decreased tear production or tear film instability, the protection of the anterior cornea may become less effective. Increased levels of oxidative and glycoxidative stress have been observed in the cornea of diabetic and keratoconus patients [45,206]. In order to counteract the oxidative stress effects, LF has been loaded into various commercial CLs by soaking in a apolactoferrin solution [44,45]. Cell viability was improved after LF release, in an epithelial cell model with induced oxidative stress. However, the maximum LF release was reached in only 1 h. Different loading strategies could help improve the release profile over time and, consequently, the clinical efficacy of the device in the treatment of corneal pathologies.

### 4.4. Posterior Segment Diseases

Macular degeneration, diabetic retinopathy, and diabetic macular edema are the most common vision-impairing diseases of the back of the eye [207]. AMD is the leading cause of elderly blindness in the United States, affecting 9.2% of individuals over 50 years old [208,209]. The pathology can be manifested under two forms, namely dry and wet AMD. Although dry AMD is more frequent, with an incidence above 85% in AMD patients, wet AMD is responsible for 90% of severe vision loss cases [209]. In the case of dry AMD, a thinning of the retinal pigment epithelium is observed, leading to blurred central vision [209]. Wet AMD is associated to retinal hemorrhage and fibrovascular tissue formation, due to an abnormal neovascularization, with the accumulation of subretinal or intraretinal fluid [210]. Cardiovascular diseases, which are linked to a higher hydrostatic pressure in the eye vessels, constitute a risk factor for the development of wet AMD [208], as well as smoking, obesity, and hereditary factors.

Most of the pathologies of the back of the eye in diabetic patients, such as DR and DME, are caused by the long term and chronic progression of microvascular damage in the retina associated with persistent hyperglycemia [211]. In its non-proliferative stage, DR is associated to an abnormal vessel permeability or the presence of microaneurysms in the capillaries. Consequently, the leaking of fluid and its accumulation in the surrounding tissue can lead to the progression of macular edema [6] and, if the subsequent swelling or thickening of the retina occurs in the fovea, DME can significantly affect vision [211]. DME can be diagnosed at any stage of DR, and its incidence increases with the progression of DR and with the duration of diabetes. Poorly controlled blood pressure, smoking, a high cholesterol level, and a reduced kidney function are also considered to be risk factors for the development of the pathology [211]. At its proliferative stage, DR can be directly related to vision impairment. In fact, neovascularization is promoted on the retinal surface due to the occlusion of capillaries, but the fragility of the newly formed capillaries leads to frequent hemorrhages. The accumulation of blood in the vitreous affects vision, while the formation of fibrotic tissue can lead to traction retinal detachment and permanent blindness [6,211]. Approximately one-third of diabetic patients are expected to develop DR, and one-tenth of these are associated to sight-threatening states [212]. Even if strict blood glucose and blood pressure control have a protective effect on the development and progression of retinopathy, they are not associated to the complete elimination of the threat.

In the past, the standard of care for proliferative DR and wet AMD was laser photocoagulation of the retinal capillaries. However, laser treatment is a destructive intervention that does not address the cause of the pathology [6,211]. The procedure is considered effective, but it is associated to side effects. For this reason, the pharmacological treatment of AMD, DR, and DME gained interest in the last decade. Alternative methods to eye drop administration have been explored for the delivery of drugs to the back of the eye due to the low penetration of many common ophthalmic drugs through the corneal epithelium and to the important drug loss caused by lacrimation [18]. The presence of the aqueous humor, the crystalline lens and the vitreous humor constitute additional barriers for drug delivery to the retina through the corneal route. An alternative path is constituted by the conjunctival-scleral route, despite the massive drug loss caused by drug absorption into the systemic circulation [10,20].

Intravitreal injections are adopted for many retinal diseases, although more invasive for the patient and associated to side effects, because they allow to deliver high doses of drug to the posterior segment of the eye [18]. Intravitreal corticosteroids, for example, have been successfully used to treat DR and DME. The intraocular administration of vascular endothelial growth factor inhibitors (anti-VEGF) or the delivery of neuroprotective agents is commonly adopted for the treatment of AMD, DR, and DME, which could be associated to laser treatment for a synergistic effect [207,212]. Monthly treatments are usually necessary due to the short half-life of drugs administered by intravitreal injections [19]. Peri-ocular administration, such as sub-tenon and subconjunctival injections, are associated to a minor risk for the patient as compared to intravitreal administration, but also to a lower efficacy [19]. In order to increase the patient comfort and decrease the risk of adverse events that are associated to frequent intravitreal injections, intravitreal implants are designed to sustain drug delivery to the posterior segment of the eye for several months [48]. By using these devices, a higher control of drug concentration in the eye over time is possible, thus avoiding the initial peak that is associated to the traditional injections.

The development of CLs and IOLs as drug depots for the delivery to the posterior segment is still at its initial stage, although some examples were reported in recent literature (Table 4). Despite the advantage of constituting a non-invasive drug-delivery strategy, drug permeability through the ocular barriers is the main issue that is associated to the use of therapeutic lenses for the back of the eye. Schultz *et al.* [31] investigated the concentration of two small-molecule drugs (prednisolone and beclomethasone) and one larger molecule (ranibizumab) in the ocular tissues of rabbits after cyclic wearing of drug-eluting CLs. Traces of all drugs were detected in the posterior segment, although not in the vitreous, which suggests a drug delivery pathway via the local vasculature. This hypothesis was supported by the study of Ross *et al.* [120], who encapsulated a dexamethasone-eluting PLGA ring into a CL: the device sustained drug release for one week *in vitro*, and it reached therapeutic drug levels in the retina *in vivo*. In fact, the drug concentration in the choroid and retina was significantly higher than in the vitreous of a rabbit model. The obtained retinal drug concentration resulted in being 200 times higher as compared to the effect of repeated administration of eye drops, and successfully inhibited retinal induced vascular leakage, thus proving the potential of therapeutic CLs for drug delivery to the posterior segment of the eye. Sharma *et al.* [108] also designed lidocaine-eluting PLGA nanoparticles, which were embedded in a collagen membrane that was attached to the CL, for drug delivery to the retina; in this case, however, *in vivo* studies were not conducted and the suitability of the system for the addressed purpose requires further investigation. A different approach was recently followed by Christopher and Chauhan [213], who proposed the design of CLs to perform iontophoresis. Iontophoresis is a promising technique in ophthalmology for the delivery of charged therapeutic molecules through the ocular barriers while using electromotive forces, but the currently available set-up is composed by two electrodes, one placed on the eye and the other on the ear or forehead. In contrast, the set-up of Christopher and Chauhan contained both of the electrodes into the CL, being less invasive for the patient. This device allowed the delivery of nile blue and fluorescein, selected as model molecules and loaded into the lenses by soaking, to the posterior segment of the eye ex vivo, paving the way for future *in vivo* experiments on drug delivery to the retina.

If the use of CLs for drug delivery to the back of the eye is still in the initial stage of investigation, then much less studies addressed the use of IOLs for the same delivery purpose. IOLs may offer the intrinsic advantage of directly delivering the drug into the aqueous humor, but their ability to deliver therapeutic drug levels to the vitreous and to the retina is still in need of proper investigation. Interestingly, a bevacizumab-eluting refillable implant [214], in order to be positioned in the peripheral lens capsule during cataract surgery, was designed for the treatment of age-related macular degeneration, thus suggesting the possibility of treating diseases of the back of the eye with drug delivery from the lens capsule and, consequently, also from IOLs. A promising result in this direction was obtained in a previously mentioned study by Eperon *et al.* [119] while using a rabbit model. Even if the target of the study was the treatment of uveitis with cyclosporine A and triamcinolone acetonide (loaded into drug-eluting reservoirs that are positioned on the IOL haptics), amounts of cyclosporine A were also detected in the retina at day 79 after surgery. This result encourages further investigation involving different therapeutics and drug loading methods. Reservoirs seem to be a very promising strategy, as the they can sustain drug release for several months; in particular, the implementation of IOL-reservoir systems addressed to the treatment of the back of the eye may potentially substitute invasive intravitreal injections for up to one year after surgery, in the case of cataract patients that are affected by pre-existing retinal diseases.

## 5. Concluding Remarks and Future Perspectives

The increasing incidence of age-related eye diseases, as well as the increasing public awareness on ocular conditions in the developed countries, determined an increase in the number of ophthalmology patients and the need for more effective and patient-friendly solutions for drug delivery to the eye. The use of drug-eluting ophthalmic lenses for the prevention and treatment of ocular pathologies, such as glaucoma, cataract, corneal, and retinal diseases, was described in this review.

Several examples of therapeutic CLs and IOLs have been developed in the last decade with promising results. The use of drug-loaded CLs can substitute the topical application of eye drops increasing the drug residence time on the cornea, the drug bioavailability, and the patient compliance. Therapeutic IOLs, which are directly implanted in the anterior chamber, are expected to perform an efficient prophylaxis for post-surgical inflammation and infection or temporarily substitute intraocular injections in case of chronic diseases.

Many examples of therapeutic CLs for the treatment of glaucoma are described in the literature. Several strategies were followed in order to improve drug loading: optimized soaking, the incorporation of functional molecules, coating, molecular imprinting, supercritical impregnation, incorporation of nanocarriers, and reservoir attachment. A prolonged release over days was obtained even by soaking in a drug solution, which constitutes the easiest drug loading method. By the incorporation of drug eluting reservoirs, drug release was extended over weeks. Despite the wide research on the topic, only a few examples of *in vivo* tests on rabbits, dogs, and monkeys were reported, with a lack of preclinical and clinical tests on glaucoma patients, which are indispensable for the future commercial application of the designed devices.

The use of CLs that are loaded with aldose-reductase inhibitors or antioxidants for the prevention of cataract in diabetic patients, especially in the young population, demonstrated, in *in vitro* studies, to be a promising approach. In the case of impaired vision and the necessity of surgical intervention, the use of therapeutic IOLs, to combine cataract surgery and the subsequent prophylaxis, has been suggested. The possibility of a dual loading of both antibiotics and anti-inflammatory drugs on IOLs constitutes an interesting solution for addressing both post-surgical infection and inflammation. Drug release for 15 days or longer can be obtained with various drugs, thus confirming the suitability of double loaded IOLs for the substitution of the current eye drops prophylaxis, usually administered for two weeks after surgery. Examples of drug eluting IOLs for the prevention of PCO were also reported and, interestingly, long-term effects were observed in rabbits, even in the case of short-term drug release. As previously stated for drug-eluting CLs, extended *in vivo* tests and subsequent pre-clinical tests are also missing for drug-eluting IOLs, despite the promising results that were obtained in rabbit models.

Keratopathies, which are usually associated to corneal epithelial defects, delayed corneal epithelial healing, tear film instability, and dry eye syndrome, can be caused by several factors, including environmental agents, inherited pathologies, ageing, diabetes, and the use of systemic or topical medications. The first-line treatment is based on the use of artificial tears to maintain a lubricated ocular surface, but a few examples of CLs releasing moisturizing agents are already present on the market. The current research is focused on the optimization of the release profiles of those agents, alone or in association with drugs for pathological conditions. In this latter case, the administration of eye drops is the standard of care, but many therapeutic CLs eluting corticosteroids and NSAIDs were reported in literature. More recently, specific drugs addressed to the treatment of keratopathy were suggested (i.e., naltrexone, growth factors, aldose reductase inhibitors, and antioxidants), and a few examples reported their sustained release by drug-eluting CLs. Preliminary clinical tests were conducted with therapeutic CLs releasing epidermal growth factors with encouraging results. However, extended experimentation is required due to the limited number of patients involved. Interestingly, the use of therapeutic CLs eluting aldose reductase inhibitors or antioxidants could have a double effect on both corneal issues and cataract prevention in the diabetic eye. In fact, the possibility of simultaneous treatment of different diseases could be an appealing objective in the design of therapeutic lenses that are addressed to diabetic patients due to the relationship between several ocular pathologies and chronic hyperglycemia.

The treatment of disorders of the posterior segment of the eye, in particular AMD, DR, and DME, is a challenging topic in ocular drug delivery. The standard of care consists in laser treatments or invasive intravitreal injections. The possibility of using therapeutic lenses for the treatment of the posterior segment of the eye is almost unexplored. Despite this, a few *in vivo* studies detected drug amounts in the retina after drug delivery through therapeutic CLs. Based on the reported cases, further research on the ocular drug delivery pathway to the posterior segment and the therapeutic efficacy of ophthalmic lenses to target tissues in the back of the eye is suggested. In fact, future *in vivo* studies could evidence the suitability of previously developed devices for drug delivery to the retina. The incorporation of innovative drug delivery methods, such as drug-eluting nanoparticles, into ophthalmic lenses could also constitute an interesting future approach for a minimally invasive sustained drug delivery to the back of the eye.

Surprisingly, the impressive efforts that were made by researchers around the world on the optimization of drug-eluting ophthalmic lenses did not yet result in the commercialization of these devices. As recently described by Lanier *et al.* [215], several reasons may be pointed out for the apparent lack of interest of the pharmaceutical industry to invest in those systems.

One common limitation of all innovative methods referred above is the need for the optimization of each specific system drug/lens. In fact, it is not possible to extrapolate the results that were obtained with a so-called model drug, because the drug release behavior and eventual alterations of the physical properties of the lenses after loading depend on the specific interactions between the drug and the polymeric matrix. For example, when using the molecular imprinting technique, the optimization of each combination polymer/monomer/crosslinker/template must be done. The control of the drug release by coating the lens strongly depends on the characteristics of the drug molecule: a very efficient coating for one drug may be inefficient for other similar drugs. Besides choosing the ideal combinations of components, it is also necessary to determine the adequate amounts of drug loaded: it has to be sufficient to ensure clinically relevant therapeutic release, but it cannot affect key aspects of the lens, namely transparency, Young modulus, ionic and oxygen permeability, wettability, and water content. In the case of the addition of other agents capable of sustaining the drug release, such as the functional monomers in the imprinting technique, vitamin E or surfactants, the preservation of the lens properties has to be ensured. The optimization of the combination of materials and loading conditions may be still more demanding when multiple drugs are needed for the treatment.

Some of the methods of preparation of drug-loaded lenses involve complex manufacturing, namely the incorporation of nanoparticles or drug reservoirs, the LbL coating, supercritical impregnation, which may be a drawback for scaling up production. Important issues, like the lack of drug stability during processing, the prevention of burst release, protein adherence, sterilization, and storage conditions, have been addressed but need more intense investigation.

In general, several innovative drug-eluting lenses have been submitted to *in vivo* studies, which demonstrated promising results; however, further studies involving the assessment of long-term safety are missing as well as extended clinical tests.

Fortunately, there are solutions for many of the technical problems described above. The minimization of burst release and protein adherence may be achieved with adequate coatings and/or optimized formulations. The storage conditions may involve immersion of the lenses in drug solutions, due to the equilibrium loading method, or keeping the lenses in dry state. Sterilization methods, which have no detrimental effects, have been proposed. Thus, the last step before commercialization needs a positive evaluation of the costs and benefits. The benefits seem to be huge, when considering that drug-eluting CLs may decrease the risks that are associated with their usual wear (keratitis, corneal erosion, dry eye syndrome, conjunctivitis) and avoid the frequent administration of eye drops for the treatment of ocular diseases, while drug-loaded IOLs may substitute the invasive intracameral and intravitreal injections. Thus, the commercialization of drug-loaded ophthalmic lenses is probable in the near future, and ongoing research on this subject continues to be relevant.

## Figures and Tables

**Table 1:** Therapeutic ophthalmic lenses developed between 2010 and 2020 suitable for the treatment of glaucoma.

Pharmacological Action	Drugs	Lens Type	Backbone Monomers	Ref.
Beta-adrenergic antagonist	Timolol	CL	HEMA	[82,102,113]
			HEMA-MAA	[86,93,103,121,135]
			HEMA-PVP	[54,93,136]
			HEMA-PC	[93]
			HEMA-DMA/GMA/Sil	[104,137]
			Sil-DMA	[55]
			Sil-DMA-MAA-PVP	[101]
			Sil-DMA-HEMA	[116]
			Sil-DMA-HEMA-PVP	[56,107,122,123]
			Sil-DMA-HEMA-PVP-PDMS	[52,101]
			Sil-PVP	[67,68,69,94]
			PDMS	[126]
			Modified PVA	[93]
			
	Betaxolol	CL	Sil-HEMA-PVP	[138]
	Puerarin	CL	HEMA-PVP-MA	[61]
Sympathomimetic agents	Brimonidine	CL	HEMA-PVP	[54]
Carbonic anhydrase inhibitors	Dorzolamide	CL	Sil-DMA-HEMA-PVP-PDMS	[52]
			
	Ethoxzolamide	CL	HEMA	[51,84]
			PVP-DMA	[85]
			
	Acetazolamide	CL	HEMA	[84]
			PVP-DMA	[85]
			HEMA-PC/MAA/PVP	[93]
			Modified PVA	[93]
			Sil-PVP	[94]
Prostaglandin analogues	Latanoprost	CL	HEMA	[38,113]
			HEMA-MAA	[124,125]
			Sil-DMA/PVP	[38]
			Sil-DMA-HEMA-PVP(-PDMS)	[38,53]
			Sil-IBM-PVP-HBM-MVA	[38]
			
	Bimatoprost	CL	Sil-DMA-HEMA	[115]
			Sil-DMA-HEMA-MAA	[83]
			Sil-DMA-HEMA-PVP(-PDMS)	[53,123]

**Table 2:** Therapeutic ophthalmic lenses developed between 2010 and 2020 suitable for the prevention of cataract and pathologies that are associated to cataract surgery.

	Pharmacological Action	Drugs	Lens Type	Backbone Monomers	Ref.
**Cataract prevention**	Aldose-reductase inhibitor	Epalrestat	CL	Sil-HEMA	[88]
	Antioxidant	Acetylcysteine	CL	HEMA-PVP	[37]
				Sil-DMA-HEMA-PVP	
				HEMA-MAA	
			
		Transferulic acid	CL	HEMA-GMA-EGPEM	[144]
**Prophylaxis after cataract surgery**	Antibiotics	Norfloxacin	IOL	HEMA	[23]
				HEMA-BEM	[60]
			
		Moxifloxacin	IOL	HEMA-MMA	[24,39,40,41,42,66,70,71,153]
				Silicone hydrogel (N/A)	[153]
				N/A	[118]
			
		Ciprofloxacin	IOL	HEMA	[26]
				HEMA-MMA	[95]
				PolyMMA	[92]
			
		Gatifloxacin	IOL	HEMA-MMA	[39]
				BMA-MMA	[96]
			
		Ampicillin	IOL	PolyMMA	[65]
			
		Levofloxacin	IOL	HEMA-MMA	[153]
				Silicone hydrogel (N/A)	[153]
	NSAIDs	Diclofenac	IOL	HEMA-MMA	[24,40,42,70,153]
				Silicone hydrogel (N/A)	[153]
			
		Ketorolac	IOL	HEMA-MMA	[24,40,70,153]
				Silicone hydrogel (N/A)	[153]
	Steroidal anti-inflammatory drugs	Dexamethasone	IOL	HEMA	[26,95]
				HEMA-MMA	[25,95]
				PolyMMA	[92]
			
		Triamcinolone acetonide	IOL	PEA-PEMA	[119]
	Immunosuppressant	Cyclosporine A	IOL	PEA-PEMA	[119]
**PCO prevention**	Anti-proliferation or apoptosis-inducing drugs	Celecoxib	IOL	Hydrophilic acrylic (N/A)	[32]
		Erufosine	IOL	Hydrophilic acrylic (N/A)	[33]
			
		Erlotinib	IOL	Hydrophilic acrylic (N/A)	[34]
				Hydrophobic acrylic (N/A)	
			
		Gefitinib	IOL	Hydrophilic acrylic (N/A)	[35]
				Hydrophobic acrylic (N/A)	
			
		Methotrexate	IOL	Hydrophilic acrylic (N/A)	[36,64]
				Hydrophobic acrylic (N/A)	[36,97]
			
		Rapamycin	IOL	PolyMMA	[72]
		Y27632	IOL	PEA-PEMA	[73]
			
		Doxorubicin	IOL	Hydrophobic acrylic (N/A)	[74]
				Hydrophobic polyester	[74,105]
			
		Indocyanine green	IOL	N/A	[77]
		MMPI	IOL	PDMS	[154]
		5-fluorouracil	IOL	PolyMMA	[106]

**Table 3:** Therapeutic ophthalmic lenses developed between 2010 and 2020 suitable for the prevention and treatment of corneal diseases.

Pharmacological Action	Drugs/Agents	Lens Type	Backbone Monomers	Ref.
Lubricating agents, moisturizing agents	HA	CL	HEMA-MAA	[176,177]
			Modified PVA	[175]
			
	HPMC	CL	HEMA(-PVP/pNIPAAm)	[179]
			Sil-DMA	[81]
			Sil-DMA-PDMS	[178]
			
	Phospholipids	CL	Silicone hydrogel (N/A)	[180,181,182]
			
	Dexpanthenol	CL	Sil-DMA-HEMA-PVP	[184]
			Sil-DMA-HEMA-PVP-PDMS	
			
	Trehalose	CL	Sil-DMA-PDMS	[178]
Osmoprotectant	Betaine	CL	Sil-DMA-HEMA-PVP	[184]
			Sil-DMA-HEMA-PVP-PDMS	
Antibiotics and anti-inflammatory drugs	Corticosteroids, NSAIDs, cyclosporine A, antibiotics	CL	Various compositions	[21,178]
Keratitis medication	Biocides	CL	HEMA-MAA-PVP	[189]
			Poly-ε-lysine	[190]
			
	Antifungals	CL	Silicone hydrogel (N/A)	[191,194]
			Poly-ε-lysine	[193]
			HEMA-PC	[194]
			HEMA-MAA	[194]
			HEMA-MAA-PVP	[189,194]
			Sil-DMA-HEMA-PVP	[194]
			Quaternized chitosan + graphene oxide	[192]
			
	Antivirals	CL	HEMA-MAA	[195,196]
Collagen photosensitizer	Riboflavin	CL	HEMA-PVP	[197,199,201]
			Sil-DMA	[201]
			PVP-MMA	[201]
Opioid antagonist	Naltrexone	CL	HEMA	[87]
Growth factor	EGF	CL	HEMA-PVP	[202]
			Sil-DMA	[43]
			PVP-MMA	[43]
			
	PDGF	CL	Modified PVA	[203]
			Sil-PVP	
			Sil-DMA	
			Sil-DMA-HEMA-PVP-PDMS	
Aldose reductase inhibitor	Epalrestat	CL	Sil-HEMA	[88]
Antioxidant	Lactoferrin	CL	Silicone hydrogels (N/A)	[44,45]

**Table 4:** Therapeutic ophthalmic lenses developed between 2010 and 2020 potentially suitable for the treatment of the back of the eye.

Pharmacological Action	Drugs/Molecules	Lens Type	Backbone Monomers	Ref.
Steroidal anti-inflammatory drugs	Prednisolone, beclomethasone	CL	PVP-MMA	[31]
	Dexamethasone	CL	HEMA-MAA	[120]
				
Anesthetic	Lidocaine	CL	N/A	[108]
				
Anti-VEGF	Ranibizumab	CL	PVP-MMA	[31]
				
-	Nile blue, fluorescein (iontophoresis)	CL	Sil-DMA-HEMA-PVP-PDMS	[213]
				
Immunosuppressant	Cyclosporine A	IOL	PEA-PEMA	[119]

## Data Availability

Data sharing not applicable.

## Glossary

**Medical terms**	
AGEs	Advanced glycation end products
AMD	Age-related macular degeneration
CLs	Contact lenses
DME	Diabetic macular edema
DR	Diabetic retinopathy
IOLs	Intraocular lenses
IOP	Intraocular pressure
PCME	Pseudophakic cystoid macular edema
PCO	Posterior capsule opacification
**Therapeutics/drugs**	
ARI(s)	Aldose reductase inhibitor(s)
EGF	Epidermal growth factor
LF	Lactoferrin
MMPI	Matrix metalloproteinases inhibitors
MTX	Methotrexate
NSAIDs	Non-steroidal anti-inflammatory drugs
NTX	Naltrexone
PDGF	Platelet-derived growth factor
TM	Timolol maleate
anti-VEGF	Vascular endothelial growth factor inhibitor
**Materials and processing terms**	
(p)HEMA	(poly)hydroxyethyl methacrylate
PBS	Phosphate buffered saline
Sil	Siloxane macromers
PGT	Propoxylated glyceryl triacylate
EGDMA	Ethylene glycol dimethacrylate
PEG	Polyethylene glycol
PLA	Polylactide
PNIPAM	Poly-*n*-isopropylacrylamide
MAA	Methacrylic acid
MA	Methyl acrylate
DMA	*N,N*-dimethylacrylamide
PLGA	Poly(lactic-co-glycolic acid)
PDMS	Poly(dimethyl)siloxane
APMA	Aminopropyl methacrylamide
MMA	Methyl methacrylate
BEM	2-butoxyethyl methacrylate
PVA	Polyvinyl alcohol
HA	Hyaluronic acid
HPMC	Hydroxypropyl methylcellulose
LbL	Layer-by-layer
NVP	*N*-vinylpyrrolidone
PVP	Polyvinylpyrrolidone
GNP	Gold nanoparticles
GMA	Glycidyl methacrylate
PC	Phosphorylcholine
IBM	Isobornyl methacrylate
HBM	2-hydroxybutyl methacrylate
MVA	*N*-methyl-*N*-vinylacetamide
PEA	Phenylethyl acrylate
PEMA	Phenylethyl methacrylate
BMA	Benzyl methacrylate
EGPEM	Ethyleneglycolphenylether methacrylate
